# CardioMEMS: the next revolution in heart failure management?

**DOI:** 10.1007/s12471-019-01356-2

**Published:** 2019-12-06

**Authors:** M. L. Handoko, A. A. van de Bovenkamp

**Affiliations:** Department of Cardiology, Amsterdam University Medical Centres, Amsterdam Cardiovascular Sciences, Vrije Universiteit, Amsterdam, The Netherlands

## Heart failure is a major clinical problem

Currently, nearly 250,000 patients suffer from heart failure in the Netherlands (1.3% of the total Dutch population) [1]. With frequent rehospitalisation for worsening of the condition and a median survival of less than 5 years (worse prognosis than most cancers!), the morbidity and mortality of heart failure are unacceptably high [1, 2] despite significant progress in heart failure treatment over the last 50 years [3]. Heart failure is a very costly condition: in 2018, around 817 million Euros (0.93% of the total health care budget) was spent on heart failure and almost half of this amount is related to hospitalisations [1]. These costs are most likely to increase in the near future: the Dutch population is aging and the survival rate of patients with an acute myocardial infarction has improved dramatically due to advances in primary percutaneous coronary interventions over the last few decades [1, 2]. Besides the high costs to society, hospitalisation for worsening heart failure has a major impact on the individual heart failure patient. Therefore, preventing (re-)hospitalisation is itself considered an important treatment goal in heart failure management [1, 2].

## Heart failure outpatient clinics in the Netherlands

What can we do to prevent heart failure hospitalisation? First of all, we need to ‘Get with the Guidelines’. For patients with heart failure with reduced ejection fraction (HFrEF; one-half to two-thirds of the total heart failure population) many effective interventions are at our disposal [2] and this arsenal may even expand over the coming years [4].

With our nurse-led heart failure outpatient clinics, we have an excellent infrastructure in the Netherlands to deliver this complex care [5]. Recent data from the Dutch CHECK-HF registry confirm that, overall, the adherence to (medical) HFrEF treatment in the Netherlands is high, when compared to the United States, for example [6, 7]. Nevertheless, there are significant unexplained institutional differences, which suggests that there is still room for improvement in HFrEF treatment [6]. Unfortunately, for patients with heart failure with preserved ejection fraction (HFpEF), no disease-modifying therapies are available: currently, symptomatic treatment with diuretics is the only salvage therapy that can be offered [2].

The heart failure outpatient clinic also allows proper patient education and easy access to medical care in case the clinical condition worsens. Self-management and early intervention may prevent hospitalisation [2, 5].

## The promise of haemodynamic heart failure telemonitoring

Even though the concept of ‘the nurse-led heart failure outpatient clinic’ is very successful, the number of heart failure hospitalisations remains high [1]. Also, patients still need to come to the hospital regularly for follow-up, which may be a burden for especially the frail and the elderly. The idea of telemonitoring to deliver heart failure care to the patient at home is appealing and not new. However, when based on heart failure symptoms and simple biometrics, like weight, heart rate and blood pressure, the concept of telemonitoring has not been very successful, as discussed by Veenis and Brugts in this issue [8]. The reason for this failure is most likely that symptoms and (non-haemodynamic) signs occur very late in the process of decompensation, so that hospitalisation is almost inevitable, despite all ‘early’ attempts to intervene.

We simply need better ways to detect early derangements in heart failure. Here, CardioMEMS may hold promise. This implantable device allows for daily recordings of pulmonary artery pressures, which are then transmitted to the heart failure clinic. Changes in these pressures are a proxy for changes in left ventricular filling pressures, generally considered one of the first things to increase when heart failure worsens. Active monitoring based on these haemodynamic measurements has been very successful and could reduce heart failure hospitalisations by half [9].

In the CHAMPION trial, heart failure medication was very frequently adjusted (both up- and down-titration) [10]. This demonstrates that the volume status in patients with heart failure (NYHA functional class III) is fluctuating continuously, requiring close monitoring. Interventions have mainly involved diuretics, but interestingly vasodilators (nitrates) have also been regularly used, which is not common practice (yet) [10]. Also, a more liberal use of pulmonary vasodilators, like sildenafil, has been suggested. A word of caution here: although it will result in a decrease in pulmonary artery pressures, left ventricular filling pressures may paradoxically increase, potentially worsening the haemodynamic situation [11].

The results of the CHAMPION trial were obtained in both HFrEF and HFpEF patients [9]. Furthermore, post-marketing studies have shown that CardioMEMS is very safe and the technique robust [12]. However, these impressive results were obtained in a context that is very different from the Dutch situation, as is further reviewed in the articles of Veenis and Brugts in this issue [8, 13]. Therefore, the results of the CHAMPION trial cannot directly be applied here. Also, it is unknown if the initial incremental costs of the CardioMEMS system (device, implantation and follow-up) are cost-effective in the Netherlands. Precisely for this reason the MONITOR-HF trial is currently being conducted: with the direct support of the Dutch Ministry of Health (*Voorlopige Toelating*), the (cost-) effectiveness of CardioMEMS will be evaluated in the Dutch health care system. Details of this multicentre randomised trial can be found in this issue [13]. Results are expected in 2022/2023.

We eagerly await the results of the MONITOR-HF trial. Hopefully CardioMEMS will fulfil its promise in revolutionising heart failure management, also for the Dutch situation.
